# How to Measure Costs and Benefits of eHealth Interventions: An Overview of Methods and Frameworks

**DOI:** 10.2196/jmir.4521

**Published:** 2015-11-09

**Authors:** Trine Strand Bergmo

**Affiliations:** ^1^ Norwegian Centre for Integrated Care and Telemedicine University Hospital of North Norway Tromsoe Norway

**Keywords:** eHealth, telemedicine, telehealth, telemonitoring, health economics, economic evaluation, cost-benefit analysis, cost-effectiveness analysis, cost-utility analysis, quality-adjusted life years (QALYs)

## Abstract

Information on the costs and benefits of eHealth interventions is needed, not only to document value for money and to support decision making in the field, but also to form the basis for developing business models and to facilitate payment systems to support large-scale services. In the absence of solid evidence of its effects, key decision makers may doubt the effectiveness, which, in turn, limits investment in, and the long-term integration of, eHealth services. However, it is not realistic to conduct economic evaluations of all eHealth applications and services in all situations, so we need to be able to generalize from those we do conduct. This implies that we have to select the most appropriate methodology and data collection strategy in order to increase the transferability across evaluations. This paper aims to contribute to the understanding of how to apply economic evaluation methodology in the eHealth field. It provides a brief overview of basic health economics principles and frameworks and discusses some methodological issues and challenges in conducting cost-effectiveness analysis of eHealth interventions. Issues regarding the identification, measurement, and valuation of costs and benefits are outlined. Furthermore, this work describes the established techniques of combining costs and benefits, presents the decision rules for identifying the preferred option, and outlines approaches to data collection strategies. Issues related to transferability and complexity are also discussed.

## Introduction

Health care costs continue to rise. An important concern for patients, clinicians, and policy makers is whether it is possible to control costs while maintaining the quality of health care services [1]. The use of information and communication technology (ICT) in health care (eHealth) is proposed as a useful tool to increase efficiency, improve access, and improve the quality of care [2,3]. In this paper, eHealth is used as an umbrella term to include telemedicine, telehealth, telecare, telemonitoring, and all other uses of ICT to provide and support the delivery of health care services at a distance [2].

Today, the use of ICT to facilitate care over distance has been investigated in almost all clinical specialties [3]. A wide number of benefits of eHealth have been reported; it can reduce time to diagnosis, improve equity of access for patients in remote areas, improve quality of life, and improve patient satisfaction [4]. In addition, eHealth has the potential to make health care workers more efficient and produce system benefits and technological spin-offs. For example, remote consultations and monitoring can deal with some of the nonurgent inquiries and thus potentially reduce office visits and other health care encounters, making the health provider more efficient [5,6]. Remote consultations can also replace or help avoid time-consuming face-to-face consultations, and for some patients, burdensome clinic visits [7]. In some situations, the use of eHealth technologies can address an unmet need for patients who might otherwise not have been in contact with their health care provider [6,8].

Another main argument for introducing eHealth services is its cost-saving potential. Some studies have found that monitoring patients at home avoids referrals and hospitalizations [9-11]. Access to relevant patient information and medical expertise to support local decisions can avoid expensive hospital visits [12]. Moreover, avoided referrals and visits have the potential to reduce patient costs associated with seeking medical help.

The cost-saving and efficiency potential of eHealth makes economic evaluation of central importance to the field [12]. Information on its costs and benefits is needed, not only to document value for money and to support decision making, but also to facilitate payment systems to support its uptake and large-scale adoption. In the absence of solid empirical evidence, key decision makers may doubt the effectiveness of eHealth, which, in turn, limits investment and its long-term integration into the mainstream health care system [13]. There is a growing frustration within health care organizations where small-scale research, pilot, or demonstration projects have failed to sustain or increase the level of use after the funding has ended [14]. Funding is often seen as the main constraint to sustaining and increasing the uptake of eHealth services. In order to secure funding, evidence of its positive effects is needed. Another constraint is the lack of health professionals’ willingness to use eHealth [15].

Numerous systematic reviews have described the evidence base as inconsistent and have called for more research [4,12,16-19]. A more recent review of reported results is more promising. They found that 23% of the papers concluded that eHealth is effective/cost-effective, and 42% were less confident about the effectiveness/cost-effectiveness. The authors suggested that these initiatives were promising, but claimed that more research is needed [20]. However, a recent large-scale telehealth evaluation—the Whole System Demonstrator project—could not establish cost-effectiveness. This evaluation was designed as a cluster randomized controlled trial with more than 3000 patients. They found no significant improvement in health outcomes, found no reductions in service use assessed over 12 months, and they reported higher costs for the telehealth option compared to usual care [21-23].

Some studies report that eHealth is cost-effective, while others cannot make this conclusion. This inconsistency and a lack of solid comparable evidence on costs and benefits can be one of the reasons for the slow uptake of eHealth interventions. Without such evidence, it is difficult to estimate the economic impact in solid business cases [24]. The decision to implement eHealth systems is generally expensive and will have an impact on different health care providers, patients, and other stakeholders. Business cases can be used to argue and document why the different stakeholders should accept and sustain eHealth. A business case is concerned with the following primary question: What do the stakeholders get out of it? To be able to answer this question, we need demonstrable evidence of costs and benefits. If large-scale eHealth implementation warrants governmental investment, this will also require demonstrable benefits for the patients, providers, and society at large [3].

The lack of economic evidence and solid business cases can further be one of the reasons why the health authorities have been hesitant to develop financial models to support eHealth interventions. Without evidence of cost-effectiveness, incentives in the form of payment systems can be misleading and encourage health managers to invest in inefficient services that cost more and are less effective than the current alternative. On the other hand, without financial models that incorporate and compensate for the resources used to provide and operate eHealth interventions, the use of eHealth services will remain limited.

Should we adopt eHealth services without solid evidence that the benefits justify its costs? It is not realistic to make one general recommendation across different services and settings. The technology, the medical field, the service provided, and local context will decide important cost parameters, such as travel costs, the need for investment in infrastructure and technologies, and the opportunity costs of health professionals, thus making it difficult to compare results across evaluations. Moreover, it is not realistic to conduct economic evaluations of all eHealth applications and services in all situations, so we need to be able to generalize from those we do conduct. This implies that we need to use appropriate techniques and evaluation methods.

This paper aims to contribute to the eHealth research field by providing a brief overview of standard economic evaluation methodology and discussing challenges in conducting cost-effectiveness analyses in eHealth. This overview might be useful in structuring and conducting future economic evaluations of eHealth. This paper outlines basic health economics principles and frameworks and describes methodological issues regarding the identification, measurement, and valuation of costs and benefits. Furthermore, it presents the established techniques of combining costs and benefits, the decision rules for identifying the preferred option, and approaches to collecting the economic data. Issues related to transferability and complexity are also discussed.

## Health Economic Evaluation: Process and Techniques

### Overview

The proper goal of any health care system is to improve the value delivered to the patients [25]. An intervention provides high value if its health benefits justify its costs. Value is measured in terms of the patient benefits achieved per euro/dollar spent. To be able to assess the value, both costs and benefits must be measured at the patient level [25]. Measuring, valuing, and comparing costs and benefits are defined within the economic evaluation framework. Economic evaluation provides information about the costs and benefits of alternative options [26]. Alternative options refer to the different ways in which health care resources can be used to improve health [27]. The alternative option in eHealth evaluations is generally usual care or normal practice. Health care costs represent the value of resources used to produce health, such as equipment, staff, and consumables. Resources outside the health system (nonhealth resources) can also be used, such as the patients’ time and the time of their families. Benefits represent all the nonresource consequences and refer to the value of changes in health outcomes for the patients. These changes can be positive and improve health, or negative and worsen health. Benefits can also include the value of security, information, and access to health services and health information.

### Evaluation Techniques

There are two main alternative techniques for aggregating costs and benefits: cost-benefit analysis (CBA) and cost-effectiveness analysis (CEA). These two techniques differ with regard to how the benefits are valued. CBA values the health outcome and other nonresource benefits in monetary terms. CBA is rarely used in health care evaluations because of the difficulty in assigning a monetary value to health outcomes [27].

In CEA, the benefits are measured as health changes. CEA aims to identify where more benefits can be produced at a lower cost or where lower costs can achieve equal benefits. In CEA, the costs in monetary terms are compared to measures of health outcomes. There are two main types of CEA: standard CEA and cost-utility analysis (CUA). In standard CEA, the costs are compared to a one-dimensional unit of effect. This could be blood glucose levels, wound size, or symptom-free days. CEA is most useful for comparing interventions that address the same health problem. For example, if the objective of using eHealth technologies in diabetes care is to reduce and stabilize blood glucose levels, it seems appropriate for the end point to measure blood glucose levels. On the other hand, it can be difficult to interpret cost-effectiveness in terms of a specific cost per reduction in blood glucose.

In CUA, the outcome is measured as "healthy years" and valued as, for example, quality-adjusted life years (QALYs). QALYs were developed to compare health gains, and they are recognized as the primary metric for measuring health status in economic evaluations [26,28]. QALYs include mortality and morbidity in one single measure [28]. The advantages of using CUA over CEA is that CUA uses one generic measure of health improvement allowing direct comparison on the same scale of different types of health effects. Furthermore, a common unit of measure—money/QALYs gained—allows comparison across different health care programs. One of the criticisms of CUA relevant for eHealth is that the benefits might extend beyond health outcomes and include access, information, waiting time, time saved, and avoidance of burdensome travels.

In both CEA and CUA, the different measures of effectiveness can be compared to the costs in a cost per unit of effect ratio, or a cost-effectiveness ratio (CER). In CEA, this could be a cost per case detected, a cost per reduction in blood glucose levels, or a cost per symptom-free day. CUA allows comparison of interventions across different types of illnesses since all CUAs report results using the same term of costs per QALY gained. Both these analyses address the question of technical efficiency and examine the effects of at least two competing alternatives within a fixed budget. In this situation, the objective is often to establish which alternative maximizes the health outcome for a given cost or minimizes the costs for a given health outcome. Table 1 compares the aggregation of consequences between the different types of analyses.

**Table 1:** Methods for aggregating costs and consequences.

Type of analysis	Aggregation of consequences
Cost-benefit analysis (CBA)	CBA measures the consequences in monetary terms expressed as a net benefit, that is, benefits minus costs. CBA answers the following question: Is the new eHealth service worthwhile?
Cost-effectiveness analysis (CEA)	CEA measures the consequences as health changes, for example, blood glucose levels, wound size, disability days avoided, and life years gained. CEA establishes which of two or more alternatives is less costly for at least as much benefit, more effective for equal or lower costs, or is more effective and more costly (in a cost per unit of effect).
Cost-utility analysis (CUA)	CUA measures the consequences as "healthy years," for example, as quality-adjusted life years (QALYs). CUA establishes which of two or more alternatives is less costly for at least as much benefit, more effective for equal or lower costs, or is more effective and more costly (in a cost per QALY).

Cost-minimization analysis (CMA) is a form of economic evaluation comparing the costs of alternative interventions that have equal effects. CMA determines the least costly alternative after the evidence indicates no important differences between the options in health outcome. CMA is generally not viewed to be an appropriate method of analysis in prospective evaluations. It is impossible to establish no difference in the health outcomes of two or more alternative options in advance, and few studies have sufficient power to show equivalence of treatments. Furthermore, the analytical focus in economic evaluations should be on the estimation of the joint density of cost and effect differences, not the outcome difference alone [29]. However, the purpose of eHealth might be to provide consultations or episodes of care. If the objective is to establish the least costly mode of delivering one specific health service, a cost-minimization analysis can be a useful framework [30,31].

Economic analyses that only focus on costs and resource use are defined as partial economic evaluations. Partial economic evaluations, such as cost analysis, can contribute useful evidence to an understanding of the cost side of eHealth interventions, but such evaluations are not well-suited to make any conclusion about cost-effectiveness. Furthermore, avoided hospitalization and travels are not benefits in this context, but costs of the alternative usual care option. If avoided hospitalizations or travels are included as a benefit (eg, in a cost per avoided hospitalization rate), the analyst must ensure that these hospitalization costs are not included as costs of the alternative option as well.

### Decision Rules in Cost-Benefit Analysis and Cost-Effectiveness Analysis

The policy question in CBA is whether one specific health intervention or program is worthwhile. If the monetary benefits exceed the costs, that is, if the results have a positive net benefit (benefit minus cost), the project or intervention is then considered worthwhile. If there are two or more worthwhile projects, the one with the highest net benefit should be chosen [32].

Within the CEA framework, deciding the preferred alternative includes assessing the costs relative to the nonmonetary outcome. A dominant strategy occurs when one alternative is producing at least as many benefits as the alternative option at less cost or is producing more benefits at equal or lower costs. In these situations, the new intervention is cost-effective, and further analysis is not needed. A dominant strategy also occurs if the new intervention costs more and is less effective.

Alternatives can be considered cost-effective even if they generate more benefits and are more costly. In order to assess if these additional benefits are worth the extra cost, a cost per unit of effect ratio, or a CER, is calculated. This ratio must be compared with ratios from an alternative option in an incremental cost-effectiveness ratio (ICER) [28]. In a health system with limited resources, choosing the alternative with the lowest ICER will generate the most value for the money.

In summary, CBA tries to answer whether a particular intervention is worthwhile, that is: Do the benefits exceed the costs? In a CEA or a CUA, the aim is to decide which of two or more alternatives is less costly for the same output or more effective for the same cost. CEA and CUA implicitly assume that one of the options will be undertaken regardless of its net benefit [9].

## Measuring Costs and Benefits of eHealth

### Overview

There is no standard recommendation on the most appropriate measure of costs and benefits for economic evaluations [33,34]. A range of different methods have been used in the literature [34]. The perspective chosen for the economic evaluation will determine what types of costs and benefits to include, for instance, whose costs and benefits are of interest. These are normative issues and must be decided in each specific evaluation. From a societal perspective, resources in all sectors (eg, hospitals, other health care institutions, municipalities, governments, and patients) are included. If the evaluation takes a health provider perspective, only health provider costs are included, and other costs, such as travel and time costs for the patients, are excluded.

### Costing

#### Overview

The costs of eHealth interventions can be divided into two broad categories: health care costs and nonhealth care costs. Direct health care costs refer to the physical health resources required to produce a specific eHealth service. Nonhealth care costs are those outside the health care sector, for example, time costs, such as production loss, lost leisure time, travel costs, and costs associated with child care.

#### Health Care Costs

Health care costs are calculated in three steps [35]. First, the health resources are identified by estimating the different categories to be included in the analysis. These can be staff, consumables, equipment, installation, readmissions, emergencies, and overhead. Second, these different resource categories are measured using appropriate physical units, for example, type of staff, the amount of time spent on different activities, type of equipment, and number of readmissions and emergencies. Third, the resources are valued using appropriate unit costs. These can be based on hospital staff salaries, marked prices, or price weights based on national tariffs or charges.

The most commonly used method for measuring and valuing health care costs is to use the resource costing method [33]. This method involves collecting health resource use data from patient charts, hospital records, or from case report forms in trials or observational studies and then multiplying service use by price weights. There is no standard method for selecting appropriate resource use and price weights. One way is to break every cost item down into its underlying components, such as laboratory tests, provider time, and drug doses (ie, microcosting). Another more common method is to identify and count health care encounters, different types of service use, or bundles of service use, such as bed days, hospital stays, outpatient consultations, and general practitioner (GP) visits (ie, gross-costing). The decision as to which bundle of resource units to include in the analysis will depend on the ease of data collection and the availability of price weights [33,36]. The resource units used must also be able to identify a true cost difference if it exists. Many evaluations use a combination of the two costing methods [37].

Price weights can be collected from the health institutions involved or previously published studies, or they can be based on national tariffs. The price weights must be relevant for the health decision context. Center-specific price weights can be obtained from the financial departments at the health institutions. National unit costs can be based on diagnosis-related groups (DRGs) or health-resource groups (HRGs), and these are often publicly available in national reference cost schedules. A more detailed description of the different costing strategies can be found elsewhere [33,34,36,38].

Implementing eHealth as part of health service delivery often incurs equipment costs. These costs can be a one-time investment cost or a monthly or yearly leasing cost. A one-time investment cost can be spread over the expected lifetime of the equipment by calculating an equivalent annual cost using a discount factor. For example, assuming a 3-year lifetime of the eHealth equipment and a 3% discount rate, the annual costs are calculated by multiplying the one-time investment costs by 0.915 (discount factor). Discount factors can be found in discount tables available online. This annuitization method is recommended as it incorporates both the depreciation and the opportunity cost of capital [26].

#### Nonhealth Care Costs

A societal perspective considers all costs, regardless of who incurs them. Nonhealth care costs are, for example, costs to social services, patients, family, and friends as informal care costs. Costs to employers as loss of production due to absence from work are also nonhealth care costs. These costs can be measured in clinical trials or observational studies. Private costs can include travel costs, out-of-pocket fees, and time costs. Time costs refer to the time patients spend seeking and receiving care and the time family members spend caring for a relative [26]. Time off work is measured as a productivity loss. Production costs are typically valued using gross wage rates. The friction cost method can also be used. Here, the basic idea is that the amount of production loss depends on the time it takes to restore the production level to where it would have been without the worker’s absence [39]. The time costs most relevant for eHealth interventions are the patients’ healthy time lost due to morbidity, assuming eHealth services improve health outcomes, and the time patients take off work to receive health care. There is no consensus on whether productivity costs should be included in cost-effectiveness analyses. Nor is there any consensus on how time costs should be valued if they are included [36,40].

### Benefit Assessment

Benefits refer to the effects that alternative interventions have on people's health. These nonresource benefits are often measured as health changes and can range from biomedical markers, to event-free time, or to more final health outcomes [38]. Outcome measures included can, for example, be blood pressure and glucose levels, cases of illness avoided, symptom-free days, successful treatments, lives saved, and life years gained. These measurements describe symptom relief and disease progression. However, the outcomes in economic evaluations should include the value patients place on the symptoms and the particular health state. The parallel is with service use on the cost side of the equation, where resource use, such as bed days and outpatient consultations, are not only counted, but also valued by measuring their costs [36]. One outcome measure that puts value on the health outcome is the QALY. The QALY includes quantity and quality of life and incorporates the valuation patients place on each health state. The QALY is the preferred outcome measure for many economists and reimbursement agencies [28,34]. The use of QALYs in telehealth studies has recently been reviewed and can be found in Bergmo [30].

Health outcomes are typically measured in clinical trials using case report forms, patient records, or patient-reported questionnaires at different time points during the trial. To estimate QALYs, the patients complete a generic health-related, quality-of-life (QoL) questionnaire with pre-existing preference weights (values). One of the most commonly used descriptive systems is the EuroQol-5D (EQ-5D) [41]. The EQ-5D is a recognized tool for describing different health states and is recommended in economic evaluation guidelines [26,28,42]. Another system is the Short-Form Health Survey-6D (SF-6D), which can be extracted from the 36-item Short-Form Health Survey (SF-36) and the 12-item Short-Form Health Survey (SF-12) [43]. These quality weights are then combined with the longevity of the improvement. This involves multiplying the quality weights for the health states developed from the questionnaires with the duration of each health state experienced by the patients. For example, 1 year in full health is one QALY; 4 years in a 0.5 quality state is two QALYs. Details on the measurement and valuation of health outcomes are fully described elsewhere [36,38,44].

Other quality-of-life instruments, such as the SF-36 and the diabetes quality-of-life (DQOL) measure are also clinically relevant and can be used to ensure that the quality of life does not differ between the alternatives under consideration. However, the usefulness of QoL measures in economic evaluations is limited since they do not rank health states according to patients’ preferences.

## Data Collection Methods

Data on costs and consequences can broadly be collected in two ways: alongside trials and observational studies, and from the existing literature [26]. New economic data can be collected alongside randomized controlled trials (RCTs), nonrandomized interventions, and observational studies. General issues in economic evaluations are common to all of these methods [26]. The RCT is often used as the gold standard for assessing the effectiveness of health interventions, but it is not always practical in eHealth research settings. Furthermore, strictly controlled trials are not well-suited for economic evaluations. Data collected alongside RCTs will provide reliable information on the particular intervention studied, but not regarding the intervention costs and how well it works for normal caseloads in usual practice. The trial context is usually very different from real-world settings, and conditions that will improve internal validity in randomized controlled trials will undermine the economic evaluation. One way to improve the usefulness of the economic evaluation is to modify the study protocol so it better reflects usual care. A naturalistic or pragmatic study design will increase the generalizability to other patients not included in the trial. A naturalistic study design is considered the gold standard for economic evaluation in health care [45].

Existing data and decision modelling is another approach that will increase the transferability of economic results across patients and settings. The data can come from clinical trials, observational studies, and meta-analyses found in the existing literature. Data can also be found in databases and administrative records. The existing evidence is brought together in a systematic way using decision models. Decision models estimate the expected costs and outcomes of different alternatives using the best available data from the literature. A well-designed model is essentially a tool that can simulate or mimic a clinical trial [46]. Models can simulate different scenarios by making explicit assumptions about the incidence, prognosis, duration, benefits, health-related quality of life, and costs. It allows one to investigate how costs and benefits might change if the values of key parameters in the model change. The purpose of modelling is not to make unconditional claims about the consequences of an intervention, but rather to reveal the relationship between assumptions and outcomes [47].

However, there are a number of valid concerns about using decision modelling [46,48]. The quality and validity of modelling studies are limited by the quality of the data used in the models. Several assumptions about the underlying disease, data, and mathematical relationship between probabilities and outcomes have to be made in modelling. These are all associated with uncertainty. The choice of one parameter over another can bias the model in favor of, or against, one particular strategy. Another challenge is that decision makers are unfamiliar with modelling. The actual calculations are often complicated and not included, and it can be difficult for decision makers to understand and thereby trust the results. To minimize uncertainty and improve transparency, the model inputs should be explicitly shown. Extensive sensitivity analysis will also help minimize uncertainty [46]. The need for, and value of, transparency is widely recognized and is cited in many guidelines [49]. See, for example, the International Society for Pharmacoeconomics and Outcomes Research (ISPOR) guidelines for good practices in decision modelling [47].

## Discussion

### Principal Findings

In this paper, I have provided a brief overview of evaluation frameworks and methodological issues regarding the collection, measurement, and valuation of costs and benefits with particular relevance to the eHealth field. The key issues outlined are as follows: useful frameworks for combining costs and benefits to support decision making, how to measure and value costs, what constitutes a useful benefit measure, how to measure the benefits, the important distinction between measurements and valuation, and approaches to data collection. The use of economic evaluation to assess the economic consequences of eHealth interventions requires adequate use of standard methodology. Transparency in reporting the methodology used is also important to ensure comparability across eHealth evaluations.

There seem to be particular challenges in conducting economic evaluations in the eHealth field. Several authors have pointed out that the heterogeneity of the eHealth field with high diversity in terms of specialties, technology, applications, objectives, context, and with many different stakeholders, can be a major challenge for economic evaluations [31,50]. The costs and effectiveness measures also tend to be multifaceted and involve a wide range of effects on patients, health care providers, and society [50]. To choose one outcome measure for the cost-effectiveness analysis can miss important benefits. It is also recognized that the measurement and valuation of some of the nonhealth consequences in eHealth research poses some difficulties. Typical benefits claimed for eHealth services that are difficult to value include improved access and a feeling of security, the value of information, and the transfer of skills. Furthermore, the QALY measure might be too incentive to capture the main outcome of interest in some of the eHealth studies [51]. When choosing an outcome measure, it is important to consider which method is most likely to be sensitive to the health change for the specific patient group included in the study. Disease-specific measures might be more sensitive to the health change an eHealth intervention is likely to produce. Disease-specific measures have, for example, been used to calculate QALYs in heart disease and cancer patients [52]. Researchers are working on developing instruments that try to measure broader outcomes within an economic evaluation framework [34,53].

It is important to consider whether the costs and consequences of interventions and their alternatives can be generalized to other settings. For example, using mobile phones to monitor symptoms in diabetes is quite different from providing specialist consultations using video conferencing or using eHealth technologies to enable home dialysis. Generalizing results from one eHealth intervention to another is problematic. The service provided and the local context will decide the most important cost parameters, such as the need for investment in infrastructure and technologies, prices, and the opportunity costs of health care professionals. If the outcome measure used is disease specific and differs from the outcome expected, this will also limit generalizability. The rapid development of this field is also a major challenge for generalizability. It is obvious that the evaluation result of a particular eHealth service is of most value in the setting where the evaluation has been conducted. However, it could be possible to generalize results from one location to another if the differences are minor. Results from an eHealth intervention within the same specialty, using similar technology, with a similar cost structure, and a generic outcome measure (or similar disease-specific measure) can most likely be generalized to a different location with some adaptation. This will require the evaluation to be transparent regarding both the methods and the context [54,55]. For example, the result will be more transparent if the quantities of resources and cost weights (unit costs) are reported separately. Furthermore, how the nonresource consequences have been measured and valued should be specified, and costs and outcomes should be reported both at baseline and at every follow-up interval.

The costing in eHealth studies is generally more straightforward than measuring and valuing health outcomes. However, it can be a challenge to find appropriate cost weights and prices. Rapidly changing technology and prices can make results less useful for decision makers. Some have called for new research strategies to deal with the rapid changes in the eHealth field [56]. The importance of including the costs of supporting the health care providers in using the eHealth interventions should also be mentioned. These can, for example, be costs of training, help desks, and change management. Adequately funding all start-up and troubleshooting costs from the beginning can make the health care clinicians more able and willing to use the new interventions.

Whether to include production loss measured as time off work is also controversial. In practice, patients may already be off work because they are retired or because of their health condition, leaving the actual production loss unchanged. Also, health visits of a shorter duration might not represent production losses at all. Some types of work can be postponed until the person is back or one’s colleagues can take over. The relevance of including time costs must be seen in relation to the perspective chosen for the evaluation. From a societal perspective, these costs are relevant, but they are not relevant if the analyst takes a health provider perspective. The time costs are important in eHealth, and one should find a way to include these costs. One way is to report production loss separately in a sensitivity analysis, leaving it to the decision makers to decide whether to include them. Another way is to report the time (hours or days) lost or gained separately without putting a value on it [26].

Whether to use trial-based data or modelling studies in economic evaluation of eHealth should be seen in relation to the objective, the role of the study, and the viewpoint of those who are expected to use the results [57]. The evidence must be relevant to the decision context. If the objective is to establish the costs and consequences of one particular eHealth service in one specific setting, the most appropriate approach is a trial-based evaluation. If, on the other hand, the decisions require more evidence than can be obtained in one single trial, data from the literature and decision modelling can be used. Whether to use a trial or modelling also depends on the existing evidence base and the quality of these data, as we need high-quality information on specific parameters in different contexts to include in decision models.

eHealth interventions are considered complex interventions by the Medical Research Council [58]. eHealth interventions are often built up from a number of components that may act interdependently, and it may be difficult to assess the many interacting components. It can also be difficult to specify what the intervention is, what is most effective, or how to replicate the intervention beyond the original study [59]. The main challenge in evaluating complex interventions is the high variability in the outcome measures. The problem of specifying the intervention is less of an issue in economic evaluation. Economic evaluations compare the value of what goes in (the resources) with what comes out (the outcomes). If you can specify the inputs and outcomes, it is not necessary to understand how the intervention works [60]. However, most eHealth interventions can be considered complex interventions implemented in complex health care systems. Complex systems pose a bigger challenge for economic evaluations. Complex systems have the tendency to change, be self-organizing, be sensitive to initial conditions, and to behave in a nonlinear fashion [61]. It is important to recognize the differences between complex interventions and complex systems and to choose appropriate evaluation methods to deal with this dual complexity. See Shiell, Hawe, and Gold for more details on economic evaluation of complex systems [60].

### Conclusions and Future Work

eHealth has been around for many years, but basic issues in relation to sustainability and large-scale eHealth systems have not been resolved. The evidence base in relation to access, quality of care, and costs are growing. However, reliable evidence of costs and benefits for decision making is still limited. The lack of evidence on costs and benefits makes it difficult to estimate the economic impact in solid business cases. The different stakeholders need to know the financial consequences of scaling up eHealth services in their local setting. Without economic evidence and solid business cases, health authorities have been hesitant to develop financial models to support eHealth services and systems. Moreover, without payment systems that cover the costs, health providers will be hesitant to invest in and provide eHealth services to their patients. Despite a limited number of large-scale services and sparse evidence that eHealth is cost-effective, interest in eHealth continues to grow. This continuous interest might be explained by the promise of eHealth to solve some of the problems in health care.

What is the best way forward when it comes to sustaining and increasing the uptake of eHealth services? First, we should continue to analyze its effectiveness and cost-effectiveness in rigorous studies. We need to improve the decision information and available data for modelling and business cases. It is not realistic to conduct economic evaluations of all eHealth applications and services in all situations, so we need to be able to generalize from those we do conduct. This implies that we have to select the most appropriate research design, cost and benefit assessment methodology, and data collection strategy in order to increase the transferability of the results. Second, we should continue to look for viable business models that explain how to deliver and sustain eHealth services at reasonable costs. We also need to decide what role eHealth should have in mainstream health care. Without funding models that ensure providers will have reliable revenue to cover the costs, eHealth will continue to be limited to small-scale pilot programs.

## Abbreviations

CBA: cost-benefit analysis
CEA: cost-effectiveness analysis
CER: cost-effectiveness ratio
CMA: cost-minimization analysis
CUA: cost-utility analysis
DQOL: diabetes quality of life
DRG: diagnosis-related group
EQ-5D: EuroQol-5D
GP: general practitioner
HRG: health-resource group
ICER: incremental cost-effectiveness ratio
ICT: information and communication technology
ISPOR: International Society for Pharmacoeconomics and Outcomes Research
QALY: quality-adjusted life year
QoL: quality of life
RCT: randomized controlled trial
SF-6D: Short-Form Health Survey-6D
SF-12: 12-item Short-Form Health Survey
SF-36: 36-item Short-Form Health Survey
